# Cost-effectiveness of twice-weekly versus once-weekly sessions of cognitive–behavioural therapy and interpersonal psychotherapy for depression at 12 months after start of treatment: randomised controlled trial

**DOI:** 10.1192/bjo.2023.548

**Published:** 2023-10-13

**Authors:** Judith E. Bosmans, Sanne J. E. Bruijniks, Mohamed El Alili, Steven D. Hollon, Frenk P. M. L. Peeters, Arnoud Arntz, Pim Cuijpers, Lotte H. J. M. Lemmens, Pieter Dingemanse, Linda Willems, Patricia van Oppen, Michael van den Boogaard, Jan Spijker, Jos W. R. Twisk, Marcus J. H. Huibers

**Affiliations:** Department of Health Sciences, Faculty of Science, Amsterdam Public Health Research Institute, Vrije Universiteit Amsterdam, The Netherlands; Department of Clinical Psychology and Psychotherapy, University of Freiburg, Germany; and Department of Clinical Psychology, Utrecht University, The Netherlands; Department of Psychology, Vanderbilt University, Tennessee, USA; Department of Clinical Psychological Science, Faculty of Psychology and Neuroscience, Maastricht University, The Netherlands; Department of Clinical Psychology, University of Amsterdam, The Netherlands; Department of Clinical Psychology, Amsterdam Public Health Research Institute, Vrije Universiteit Amsterdam, The Netherlands; Division of Affective Disorders, Mental Health Care Altrecht, The Netherlands; Department of Mood Disorders, GGZ Oost-Brabant, The Netherlands; Department of Research and Innovation, GGZ InGeest Specialized Mental Health Care, The Netherlands; and Department of Psychiatry, Amsterdam University Medical Centers, Amsterdam Public Health Research Institute, Vrije Universiteit Amsterdam, The Netherlands; PsyQ, Parnassia Groep, The Netherlands; Depression Expertise Centre, Pro Persona Mental Health Care, The Netherlands; and Behavioral Science Institute, Radboud University, The Netherlands; Department of Epidemiology and Biostatistics, Amsterdam University Medical Centers, Amsterdam Public Health Research Institute, Vrije Universiteit Amsterdam, The Netherlands

**Keywords:** Depressive disorders, cost-effectiveness, individual psychotherapy, out-patient treatment, randomised controlled trial

## Abstract

**Background:**

Cost-effective treatments are needed to reduce the burden of depression. One way to improve the cost-effectiveness of psychotherapy might be to increase session frequency, but keep the total number of sessions constant.

**Aim:**

To evaluate the cost-effectiveness of twice-weekly compared with once-weekly psychotherapy sessions after 12 months, from a societal perspective.

**Method:**

An economic evaluation was conducted alongside a randomised controlled trial comparing twice-weekly versus once-weekly sessions of psychotherapy (cognitive–behavioural therapy or interpersonal psychotherapy) for depression. Missing data were handled by multiple imputation. Statistical uncertainty was estimated with bootstrapping and presented with cost-effectiveness acceptability curves.

**Results:**

Differences between the two groups in depressive symptoms, physical and social functioning, and quality-adjusted life-years (QALY) at 12-month follow-up were small and not statistically significant. Total societal costs in the twice-weekly session group were higher, albeit not statistically significantly so, than in the once-weekly session group (mean difference €2065, 95% CI −686 to 5146). The probability that twice-weekly sessions are cost-effective compared with once-weekly sessions was 0.40 at a ceiling ratio of €1000 per point improvement in Beck Depression Inventory-II score, 0.32 at a ceiling ratio of €50 000 per QALY gained, 0.23 at a ceiling ratio of €1000 per point improvement in physical functioning score and 0.62 at a ceiling ratio of €1000 per point improvement in social functioning score.

**Conclusions:**

Based on the current results, twice-weekly sessions of psychotherapy for depression are not cost-effective over the long term compared with once-weekly sessions.

With a worldwide 12-month prevalence of 4–14% and lifetime prevalence of 11–20%, depression is the largest contributor to the global burden of non-fatal disease.^1–5^ Compared with healthy individuals, individuals with depression experience physical problems such as feeling tired and less energised, as well as emotional problems that interfere with daily activities.^5^ Depression is also associated with a higher use of medical health services and loss of productivity resulting from increased absence or underperformance at work.^4^ To reduce the burden of depression for the individual and for society, cost-effective treatments are needed. Studies have suggested that different forms of psychotherapy, such as behavioural activation, cognitive–behavioural therapy (CBT) and interpersonal psychotherapy (IPT), can be cost-effective compared with treatment as usual in adults with depression.^6^ In addition, certain forms of psychotherapy might be more cost-effective than others. For example, implementation of a simpler behavioural activation programme by less experienced mental health workers was shown to be cost-effective compared with CBT, which is more dependent on the skills of the therapist, and so is delivered by more expensive and better trained therapists.^7^ However, findings with regard to the cost-effectiveness of treatments for depression remain mixed, and further studies are necessary to reduce the economic burden of depression.^6^

One way of changing the delivery of psychotherapy to increase cost-effectiveness is by increasing initial session frequency, but keeping the total number of sessions constant. Recently, we showed that increasing the session frequency from one session to two sessions a week is clinically more effective than one session a week at the end of treatment (i.e. after 6 months).^8^ We hypothesise that a higher initial session frequency is not only more effective, but also leads to lower societal costs than a lower frequency, because patients may be able to go back to work more quickly and there may be a reduction in use of mental healthcare services in the long term. The aim of the present study was to investigate the cost-effectiveness, from a societal perspective, of twice-weekly versus once-weekly sessions of psychotherapy (CBT or IPT) for depression at 12 months after the start of treatment.

## Method

### Design

The economic evaluation was conducted alongside a multicentre randomised controlled trial (2 × 2 factorial design) comparing once-weekly versus twice-weekly sessions of psychotherapy (CBT or IPT) for depression with a 24-month follow-up. Data presented in this article concern the cost-effectiveness at 12 months after the start of treatment, and were previously published as a chapter in a PhD thesis.^9^ Patients were randomly assigned to four conditions, using an allocation scheme generated by an independent researcher: (a) CBT sessions twice a week (*n* = 49), (b) CBT sessions once a week (*n* = 49), (c) IPT sessions twice a week (*n* = 47) and (d) IPT sessions once a week (*n* = 55). Block randomisation was used stratified for depression severity (high: Beck Depression Inventory-II (BDI-II) score ≥29; low: BDI-II score ≤28) and treatment site. Blinding of patients and therapists was not possible because of the nature of the intervention. Further details about the study design can be found elsewhere.^10^

The authors assert that all procedures contributing to this work comply with the ethical standards of the relevant national and institutional committees on human experimentation and with the Helsinki Declaration of 1975, as revised in 2008. All procedures involving human patients were approved by VU Medical Centre Amsterdam (approval number 2014.337). The study is registered with The Netherlands Trial Register (https://www.trialregister.nl; trial registration number NTR4856). Participant registration took place from October 2014 to April 2018. All adult participants provided written informed consent to participate in this study.

### Participants

Patients were adult out-patients referred to one of nine Dutch specialised mental healthcare centres located across The Netherlands. Inclusion criteria were as follows: (a) a primary diagnosis of DSM-IV or DSM-5 major depressive disorder (including chronic depression) or DSM-5-based persistent depressive disorder as confirmed by the Structural Clinical Interview for DSM-IV Axis I disorders (SCID-I^11^) or the Mini-International Neuropsychiatric Interview Plus (MINI-Plus^12^), (b) aged 18 to <65 years, (c) sufficient knowledge of the Dutch language, (d) pre-treatment score of ≥20 on the BDI-II^13^ and (e) access to internet facilities (some assessments were online). Exclusion criteria were as follows: (a) starting antidepressants or dosage change <3 months before baseline; (b) acute risk of suicide; (c) DSM-IV or DSM-5 diagnosis of substance use disorders; (d) presence of a DSM-IV or DSM-5 diagnosis of a cluster A or B personality disorder, as evaluated by a clinician during the intake with or without a structured interview and (e) having received more than five sessions of adequate CBT or IPT in the previous year (clinician-evaluated at intake).

### Interventions

The same treatment manuals were used for both CBT and IPT regardless of session frequency. CBT was based on the manual by Beck et al^14^ and IPT was based on the manual by Klerman et al.^15^ Both CBT and IPT consisted of 12–20 face-to-face 45-min sessions, with the total number depending on patient progress. Participants randomised to the condition with twice-weekly sessions received 16 sessions during the first 8 weeks of treatment, and four sessions during the final 8 weeks (up to 20 sessions over a period of 16 weeks). Patients randomised to the condition with once-weekly sessions received 16 sessions during the first 16 weeks of treatment, and four sessions during the final 8 weeks (up to 20 sessions over a period of 24 weeks).

### Clinical outcomes

Severity of depressive symptoms was measured with the BDI-II at baseline, and at 3, 6, 9 and 12 months after baseline.^13^ The BDI-II is a 21-item self-report instrument assessing depressive symptoms over the past 2 weeks, with higher scores indicating more severe depression.^16^ Quality of life was measured at baseline, and 3, 6, 9 and 12 months of follow-up, using the five-level version of the EuroQol (EQ-5D-5L^17^) and the RAND 36-Item Health Survey (RAND-36^18^). EQ-5D-5L health states were converted to utility values with the Dutch EQ-5D-5L tariff^17^. The utility values were then used to calculate quality-adjusted life-years (QALY), using an area-under-the-curve approach. Specifically, we multiplied the average utility value of two measurements with the time in years that had passed between these two measurements. Next, the QALY estimates for each time period were summed to calculate the total number of QALYs over 12 months. Based on the RAND-36, scores for two subdomains were calculated: the physical functioning score (PFS) and the social functioning score (SFS). These scores were transformed to a 0–100 scale, with higher scores indicating better quality of life.

### Cost outcomes

Costs (given in Euros, index year 2021) were measured from a societal perspective, using a specifically adapted version of the Trimbos/iMTA Questionnaire for Costs associated with Psychiatric Illness (TiC-P)^19^ at baseline, and 3, 6, 9 and 12 months of follow-up. Thus, discounting was not necessary. Costs included healthcare costs, informal care costs and lost productivity costs. Lost productivity costs included absenteeism from paid and unpaid work, and presenteeism. Presenteeism is defined as coming to work despite having health problems, resulting in less efficiency when working. Use of healthcare services was valued with Dutch standard costs when available.^20^ If not, tariffs of professional organisations or professionals themselves were used. Medication was valued with prices from the Dutch National Healthcare Institute (www.medicijnkosten.nl). Absenteeism from paid work was valued with the friction cost approach. The friction cost approach assumes that sick employees are replaced after a certain time period (the friction period), after which productivity is restored to the old level. A friction period of 12 weeks was used, and absenteeism was valued using gender-specific average wage rates for the Dutch population.^20^ Participants registered their level of efficiency when present at their work with health complaints (i.e. the efficiency score). Lost productivity was calculated as (1 − efficiency score) × number of days with health complaints × hours per day. This was then valued using gender-specific wage rates. Absenteeism from unpaid work was valued using a shadow price for a legally employed cleaner.

### Sample size

Based on a meta regression analysis that indicated an increase from one to two sessions per week increased the effect size *g* = 0.45,^21^ we estimated the post-treatment effect size to be around 0.45. Taking a 20% drop-out rate into account, based on an alpha of 0.05 and power of 0.80, a sample size of 200 patients was needed.

### Data analyses

All analyses compared twice-weekly sessions with once-weekly sessions of psychotherapy, and were conducted according to the intention-to-treat principle. Missing clinical outcomes and cost data were estimated by using multiple imputation by chained equations, with predictive mean matching to account for the skewed distribution of costs.^22^ An imputation model was created that included baseline characteristics differing between treatment groups, baseline characteristics differing between participants with and without complete follow-up, baseline characteristics related to missing outcomes and all variables included in the analysis models. The number of imputations was increased until the fraction of missing information was <5%. The imputed data-sets were analysed separately as described below, and results were subsequently pooled according to Rubin's rules.^23^

To account for the 2 × 2 factorial design, we adjusted the analysis models for type of psychotherapy (CBT or IPT). Differences in BDI-II scores over time were estimated with a fixed-effects longitudinal mixed model, with patient and time as hierarchical levels. Baseline BDI-II value was included as a covariate in the longitudinal mixed model. Differences in PFS, SFS, QALYs and costs after 12 months were estimated with a linear regression model. To account for the skewed distribution of costs, statistical uncertainty was estimated by using bias-corrected bootstrapping with 5000 replications.

Incremental cost-effectiveness ratios (ICERs) were calculated by dividing the pooled difference in costs by the pooled difference in effects. Bias-corrected bootstrapping (5000 replications) was used to estimate the statistical uncertainty surrounding the ICERs. The proportion of bootstrapped cost-effect pairs in each quadrant of the cost-effectiveness plane was estimated across all imputed data-sets to show uncertainty surrounding the ICER. In addition, cost-effectiveness acceptability curves were estimated that show the probability that twice-weekly sessions are cost-effective in comparison with once-weekly sessions for different ceiling ratios. The probability of cost-effectiveness was determined with the net monetary benefit framework, using pooled estimates of net monetary benefit and the s.e. The ceiling ratio is the amount of money that society is willing to invest to gain one unit of improvement in a specific effect outcome. For outcomes like the BDI-II, PFS and SFS, no ceiling ratios have been defined. For QALYs, the National Institute for Health and Care Excellence in the UK established ceiling ratios between £20 000 and £30 000 per QALY gained (between €23 000 and €34 000 per QALY gained). The utility scores for having moderate and severe depression have been estimated at 0.52 and 0.39, respectively, resulting in a disease burden of 0.48 and 0.61 (i.e. 1 – utility score), respectively.^24^ In The Netherlands, ceiling ratios between €20 000 and €50 000 per QALY gained are used for health conditions with a disease burden between 0.41 and 0.7.

### Sensitivity analyses

Four sensitivity analyses were conducted to assess the robustness of the results. The first sensitivity analysis concerns an analysis from the healthcare perspective, meaning that only healthcare costs were included. In the UK for example, reimbursement decisions are made from the healthcare perspective. In the second sensitivity analysis, lost productivity costs owing to absenteeism from paid work were valued with the human capital approach. The human capital approach assumes that lost productivity costs are generated during the full period of absenteeism and is internationally more commonly used than the friction cost approach. For the third sensitivity analysis, outliers for costs were recoded as missing and imputed in the multiple imputation procedure. Outliers were arbitrarily defined as participants generating total societal costs of €10 000 or more at any of the four time points at which cost questionnaires were administered. In the fourth sensitivity analysis, total societal costs over 12 months after the start of treatment were adjusted for total societal costs in the 3 months before start of treatment.

## Results

Recruitment took place between November 2014 and January 2018. In total, 96 patients were randomised to twice-weekly sessions and 104 patients were randomised to once-weekly sessions. A description of the baseline characteristics is shown in Table 1. Follow-up on outcomes and costs was obtained from 145 (72.5%) participants at 3 months of follow-up, 153 (76.5%) participants at 6 months of follow-up, 140 (70%) participants at 9 months of follow-up and 138 (69%) participants at 12 months of follow-up (see the Consolidated Standards of Reporting Trials flow chart in Supplementary Appendix 1 available at https://doi.org/10.1192/bjo.2023.548). Participants with complete follow-up for costs and effects had higher physical functioning (as indicated by the PFS) and were more highly educated than participants without complete follow-up.

**Table 1:** Baseline characteristics stratified by treatment group

	Twice-weekly sessions (*n* = 96)	Once-weekly sessions (*n* = 104)
Female, *n* (%)	57 (59.4)	66 (63.5)
Age, years, mean (s.d.)	39.55 (12.26)	36.28 (12.10)
Highest completed education, *n* (%)		
Low	11 (11.5)	10 (9.6)
Medium	52 (54.2)	50 (48.1)
High	33 (34.4)	44 (42.3)
Partner, yes, *n* (%)	39 (40.6)	33 (31.7)
Current job, yes, *n* (%)	57 (59.4)	58 (55.8)
Born in The Netherlands, *n* (%)	74 (77.1)	85 (81.7)
Chronic depression, *n* (%)	45 (46.9)	48 (46.2)
Baseline BDI-II score, mean (s.d.)	34.80 (10.50)	34.61 (9.48)
Baseline RAND-36 PFS, mean (s.d.)	71.1 (24.9)	70.0 (27.5)
Baseline RAND-36 SFS, mean (s.d.)	36.1 (21.6)	31.1 (22.4)
Baseline EQ-5D-5L utility score, mean (s.d.)	0.49 (0.28)	0.44 (0.31)

BDI-II, Beck Depression Inventory-II; RAND-36, RAND 36-Item Health Survey; PFS, physical functioning score; SFS, social functioning score; EQ-5D-5L, EuroQol five-level version.

### Effects

The course of BDI-II scores over 12 months is shown in Table 2 and Supplementary Appendix 2. BDI-II scores in the twice-weekly group decreased more over the first 6 months of follow-up than in the once-weekly group, and were similar after 9 months; however, at 12 months of follow-up, the BDI-II scores in the twice-weekly group were again lower than in the once-weekly group. The overall average decrease in BDI-II scores over 12 months of follow-up in the twice-weekly group was larger than in the once-weekly group (mean difference −1.36), but this difference was not statistically significant (95% CI −4.83 to 2.11). Participants in the twice-weekly group gained on average 0.017 QALYs compared with the once-weekly group, but this difference was not statistically significant (95% CI −0.053 to 0.088). Differences in PFS and SFS after 12 months between groups were small and not statistically significant (Table 2).

**Table 2:** Mean (s.e.) clinical outcomes and costs 12 months after start of treatment, stratified by session frequency and differences in costs and effects after multiple imputation

	Twice-weekly sessions (*n* = 96)	Once-weekly sessions (*n* = 104)	Mean difference (95% CI)^a^^,^^b^
Clinical outcomes			
BDI-II score^c^		−1.36 (−4.83 to 2.11)^c^	
Baseline	34.80 (1.07)	34.62 (0.93)	
3 months	24.75 (1.98)	26.81 (2.23)	
6 months	22.54 (2.17)	25.09 (2.21)	
9 months	22.75 (2.15)	22.63 (2.54)	
12 months	20.49 (2.97)	22.53 (2.53)	
EQ-5D-5L			
Baseline	0.49 (0.028)	0.44 (0.030)	
3 months	0.58 (0.045)	0.49 (0.038)	
6 months	0.58 (0.043)	0.57 (0.044)	
9 months	0.59 (0.042)	0.61 (0.048)	
12 months	0.57 (0.047)	0.63 (0.057)	
QALY	0.57 (0.025)	0.55 (0.026)	0.017 (−0.053 to 0.088)
PFS at 12 months	75.51 (4.26)	78.01 (3.49)	−2.59 (−14.75 to 9.58)
SFS at 12 months	60.70 (4.52)	56.36 (4.46)	4.33 (−10.72 to 19.39)
Cost outcomes			
Total healthcare costs	10 585 (1260)	6667 (659)	3901 (1939–6345)
Primary care	900 (101)	797 (80)	102 (−93 to 306)
Secondary care	2052 (476)	2000 (494)	48 (−808 to 1204)
Mental healthcare	5730 (1072)	2178 (399)	3541 (2068–5375)
Medication	82 (13)	69 (13)	13 (−13 to 41)
Intervention	1779 (58)	1585 (67)	193 (22–373)
Supportive care	42 (23)	37 (18)	3 (−32 to 79)
Informal care costs	2885 (304)	3220 (298)	−339 (−1082 to 391)
Lost productivity costs	5028 (516)	6321 (841)	−1286 (−2789 to 1524)
Absenteeism unpaid work	2733 (285)	3121 (301)	−390 (−1114 to 316)
Presenteeism paid work	1684 (392)	2817 (726)	−1129 (−2305 to −30)
Absenteeism paid work	611 (108)	381 (84)	233 (26–458)
Total societal costs	18 498 (1541)	16 208 (1306)	2276 (−756 to 5671)

BDI-II, Beck Depression Inventory-II; EQ-5D-5L, EuroQol five-level version; QALY, quality-adjusted life-year; PFS, physical functioning score; SFS, social functioning score.

a. 95% confidence intervals estimated with bias-corrected and accelerated bootstrapping.

b. All analyses adjusted for type of psychotherapy (cognitive–behavioural therapy or interpersonal psychotherapy).

c. Overall effect over time, i.e.12 months after start of treatment (95% CI) corrected for BDI-II score at baseline.

### Costs

Table 2 also shows costs at 12 months of follow-up in the two study groups. On average, participants in the twice-weekly group (16.5 sessions in total) received 1.8 psychotherapy sessions more than in the once-weekly group (14.7 sessions in total), resulting in statistically significantly higher intervention costs in the twice-weekly group (mean difference €193, 95% CI 20–338). Mental and total healthcare costs in the twice-weekly group were also statistically significantly higher than in the once-weekly group. This was caused by the presence of a higher number of outliers in the twice-weekly group (six) compared with the once-weekly group (three). Four of the six outliers in the twice-weekly group were caused by high mental healthcare costs (e.g. because of admission to a psychiatric hospital). However, total lost productivity costs in the twice-weekly group were statistically non-significantly lower than in the once-weekly group, because of lower presenteeism costs in the twice-weekly group. Supplementary Appendix 3 shows the distribution of the different cost categories within total societal costs. Overall, total societal costs over 12 months in the twice-weekly group were higher than in the once-weekly group (mean difference €2065), although this difference was not statistically significant.

### Cost-effectiveness

**Fig. 1:**
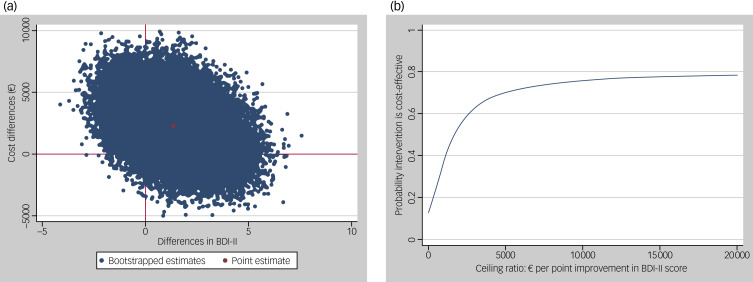
(a) Cost-effectiveness plane for the difference in BDI-II score at 12 months. (b) Cost-effectiveness acceptability curve for the difference in BDI-II score at 12 months. BDI-II, Beck Depression Inventory-II.

The results of the cost-effectiveness analyses are shown in Table 3. For the BDI-II, the ICER was −1670, indicating that to gain one point of improvement in BDI-II score in the twice-weekly group, on average €1670 per person needs to be invested compared with the once-weekly session group. The majority (71%) of bootstrapped cost-effect pairs is in the north-east quadrant of the cost-effectiveness plane (Fig. 1a). The cost-effectiveness acceptability curve (Fig. 1b) shows that when society is not willing to pay any money for one point of improvement in BDI-II score (i.e. at a ceiling ratio of €0 per point improvement in BDI-II score), the probability that twice-weekly sessions are cost-effective in comparison with once-weekly sessions is 0.13. This increases to 0.37 at a ceiling ratio of €1000 per point improvement in BDI-II score.

**Table 3:** Results of the cost-effectiveness analyses (main analysis and sensitivity analyses) for two sessions per week compared with one session per week

Outcome	Difference in costs (95% CI)^a^^,^^b^	Difference in effects (95% CI)^a^^,^^b^	ICER	Cost-effectiveness plane			
				North-east	South-east	South-west	North-west
Main analysis: societal perspective							
BDI-II^c^	2276 (−824 to 5611)	−1.36 (−4.39 to 1.66)	−1670	71%	12%	1%	16%
QALYs	2276 (−766 to 5690)	0.017 (−0.053 to 0.088)	130 693	60%	10%	3%	27%
PFS	2276 (−766 to 5690)	−2.59 (−14 to 8.82)	−878	26%	6%	7%	61%
SFS	2276 (−766 to 5690)	4.33 (−9.66 to 18.33)	525	64%	10%	3%	23%
Sensitivity analysis 1: healthcare perspective							
BDI-II^c^	3901 (1849–6227)	−1.36 (−4.39 to 1.66)	−2863	83%	0%	0%	17%
QALYs	3540 (1929–6345)	0.017 (−0.053 to 0.088)	224 063	69%	0%	0%	31%
PFS	3540 (1929–6345)	−2.59 (−14 to 8.82)	−1507	32%	0%	0%	68%
SFS	3540 (1929–6345)	4.33 (−9.66 to 18.33)	900	73%	0%	0%	26%
Sensitivity analysis 2: human capital approach							
BDI-II^c^	3976 (−9 to 8175)	−1.36 (−4.39 to 1.66)	−2918	78%	5%	0%	17%
QALYs	3976 (51–8238)	0.017 (−0.053 to 0.088)	228 387	65%	4%	1%	30%
PFS	3976 (51–8238)	−2.59 (−14 to 8.82)	−1535	31%	2%	3%	64%
SFS	3976 (51–8238)	4.33 (−9.66 to 18.33)	917	69%	4%	1%	26%
Sensitivity analysis 3: outliers recoded as missing							
BDI-II^c^	1599 (−723 to 4497)	−1.29 (−3.82 to 1.24)	−1237	76%	10%	1%	13%
QALYs	1599 (−683 to 4125)	0.020 (−0.053 to 0.093)	81 329	63%	8%	2%	27%
PFS	1599 (−683 to 4125)	−2.62 (−10 to 4.89)	−611	22%	3%	7%	68%
SFS	1599 (−683 to 4125)	2.81 (−9.13 to 14.74)	570	59%	8%	3%	3%
Sensitivity analysis 4: adjusted for baseline societal costs							
BDI-II^c^	2329 (−746 to 5645)	−1.36 (−4.39 to 1.66)	−1709	71%	12%	0%	17%
QALYs	2331 (−724 to 5711)	0.019 (−0.051 to 0.089)	125 303	61%	9%	3%	27%
PFS	2330 (−721 to 5713)	−2.83 (−13.96 to 8.30)	−824	25%	5%	7%	63%
SFS	2332 (−721 to 5717)	4.37 (−9.32 to 18.07)	533	65%	9%	3%	23%

ICER, incremental cost-effectiveness ratio; BDI-II, Beck Depression Inventory-II; QALY, quality-adjusted life-year; PFS, physical functioning score; SFS, social functioning score.

a. 95% confidence intervals estimated with bias-corrected and accelerated bootstrap.

b. All analyses adjusted for type of psychotherapy (cognitive–behavioural therapy or interpersonal psychotherapy).

c. Overall effect over time corrected for BDI-II score at baseline.

For QALYs, the ICER was 130 693, meaning that to gain one QALY in the twice-weekly group, on average €130 693 should be invested compared with the once-weekly group. At ceiling ratios of €0, €20 000 and €50 000 per QALY gained, the probabilities that twice-weekly sessions are cost-effective in comparison with once-weekly sessions are 0.13, 0.18 and 0.30, respectively.

For the PFS, the ICER was −878, indicating that effects were smaller and total societal costs higher in the twice-weekly group compared with the once-weekly group.

For the SFS, the ICER was 525, meaning that to gain one point of improvement in SFS, an investment of €525 is needed for twice-weekly sessions compared with once-weekly sessions. At ceiling ratios of €0 and €1000 per point improvement in SFS, the probabilities that twice-weekly sessions are cost-effective compared with once-weekly sessions are 0.13 and 0.62, respectively.

### Sensitivity analyses

In the first two sensitivity analyses (healthcare perspective and human capital approach), the difference in total costs between twice-weekly sessions and once-weekly sessions increased compared with the main analysis (Table 3). The probability that twice-weekly sessions are cost-effective compared with once-weekly sessions was 0.01 at a ceiling ratio of €0 per additional unit of effect. This increased to 0.17 at a ceiling ratio of €1000 per point improvement in BDI-II score, 0.12 at a ceiling ratio of €50 000 per QALY gained, 0.17 at a ceiling ratio of €1000 per point improvement in PFS and 0.54 at a ceiling ratio of €1000 per point improvement in SFS.

When using the human capital approach, the difference in total societal costs increased to €3976, which was statistically significant. This increase is because there were relatively more people with long-term absenteeism from paid work during follow-up in the twice-weekly session group compared with the once-weekly session group. The probability of cost-effectiveness at a ceiling ratio of €0 per additional unit of effect was 0.06. This increased to 0.24 at a ceiling ratio of €1000 per point improvement in BDI-II score, 0.19 at a ceiling ratio of €50 000 per QALY gained, 0.16 at a ceiling ratio of €1000 per point improvement in PFS and 0.54 at a ceiling ratio of €1000 per point improvement in SFS.

The third sensitivity analysis, where outliers were recoded as missing before performing multiple imputation, resulted in a substantial decrease in the societal cost difference from €2276 to €1599, compared with the main analysis. However, effect estimates also changed slightly. As a result, the probability of cost-effectiveness at a ceiling ratio of €0 per additional unit of effect did not change much compared with the main analysis (i.e. 0.12 regardless of the outcome measure). For BDI-II score and QALYs gained, the probabilities of cost-effectiveness increased (0.43 at €1000 per point improvement in BDI-II score and 0.40 at €50 000 per QALY gained), whereas for the PFS and SFS the probabilities decreased (0.16 at €1000 per point improvement in PFS and 0.57 at €1000 per point improvement in SFS). Detailed outcomes can be found in Supplementary Appendix 4.

In the fourth sensitivity analysis (adjustment for societal costs at baseline), the difference in total societal costs between groups 12 months after start of treatment (€2329) was slightly higher than in the main analysis (€2276). Overall, results were similar to the main analysis.

## Discussion

### Main findings and explanation of findings

Our study showed that differences in clinical outcomes after 12 months between twice-weekly and once-weekly psychotherapy sessions were modest. In addition, total societal costs at 12 months in the twice-weekly group were statistically non-significantly higher than in the once-weekly session group. Although no ceiling ratios have been established for clinical outcomes other than QALYs, the probability of cost-effectiveness of twice-weekly sessions compared with once-weekly sessions is low at ceiling ratios that may be considered acceptable for depressive symptoms, and physical and social functioning. For QALYs gained, the probability of cost-effectiveness was between 0.18 and 0.30 at commonly accepted ceiling ratios of between €20 000 and €50 000 per QALY gained. This indicates that even if society is willing to pay large amounts of money, it is uncertain that twice-weekly sessions are cost-effective compared with once-weekly sessions.

The previous analyses of the 6-month effectiveness data showed that twice-weekly sessions led to a statistically significant greater and faster reduction in depressive symptoms compared with once-weekly sessions.^8^ After 12 months, the reduction in depressive symptoms in the twice-weekly group was still larger than in the once-weekly group, but this difference was no longer statistically significant. Although the analytical approach in the current study differed from the analysis at 6 months of follow-up (use of multiple imputation and a different analysis model), we think it is likely that this is a true effect, since both analyses show that the difference in BDI-II score was largest at 6 months. Thus, although twice-weekly sessions of psychotherapy for depression result in faster recovery for patients, this is not cost-effective over the long term based on the current results.^8^

### Comparison with the literature

Comparison of our results to other studies is difficult because, to the best of our knowledge, this is the first cost-effectiveness study comparing twice-weekly with once-weekly psychotherapy sessions for depression. However, when comparing our results to the results of the economic evaluations on different forms of psychotherapy, mean total societal costs per participant in this economic evaluation were relatively high.^6,25^ This may be a reflection of the severe depressive symptoms and substantial chronicity at baseline in our sample compared with the samples included in these previous studies.^6,25^ Also, at 12 months of follow-up, the mean BDI-II scores (20.49 and 22.53 in the twice-weekly and once-weekly groups, respectively) indicate that most patients still have from moderate depression (i.e. BDI-II score between 19 and 29) at the end of the 12-month follow-up, which underlies the severity of depression in this sample of patients.

### Strengths and limitations

Our randomised controlled trial had a pragmatic design, meaning that we tried to resemble actual clinical practice as much as possible. Thus, a main strength of the current study is the generalisability of the findings to other settings. A second strength of the study is that a wide range of outcomes was assessed: severity of depressive symptoms, physical and social functioning and QALYs gained. Finally, costs were measured from the broadest perspective possible, the societal perspective. This means that it is possible to identify potential cost shifts between sectors; for example, hypothetically more intensive psychotherapy treatment may lead to higher costs (healthcare system costs), but these costs may be offset by lower absenteeism resulting in lower societal costs. For the current study, this is important, because we expected *a priori* that healthcare costs would be similar between groups, but that participants in the twice-weekly group would recover sooner than participants in the once-weekly group, and would therefore have lower lost productivity costs. There are also several limitations that need to be mentioned. First, the quality of the delivered psychotherapy sessions ranged widely, from poor to good.^8^ It may be possible that the effects of increased session frequency at the start of treatment are larger when the quality of the delivered psychotherapy is higher. Second, the number of participants with one or more missing questionnaires was considerable for both costs and effects. To account for this, we used multiple imputation, which is generally considered the most appropriate method to impute missing data.^26,27^ Finally, the difference in healthcare costs was highly influenced by a few outliers in the twice-weekly group who had very high mental healthcare costs resulting from intensive mental care treatment including hospital admission, and this effect may be inflated by the multiple imputation procedure because the multiple imputation procedure was stratified by intervention group. Considering the more positive effect on clinical outcomes, we consider this a random finding.

### Implications for research and clinical practice

From a patient perspective, increasing the frequency of psychotherapy sessions per week leads to faster response.^8^ However, there was a significant difference in healthcare costs between the twice and once-weekly groups that was mainly driven by higher mental healthcare costs in the twice-weekly group compared with the once-weekly group, caused by several outliers in the twice-weekly group. Although this difference in healthcare costs was partly offset by statistically non-significantly lower lost productivity costs in the twice-weekly group compared with the once-weekly session group, from a societal perspective, twice-weekly sessions cannot be considered cost-effective based on the current results. Considering the beneficial effect for patients because of faster response to treatment, it is important to perform more studies to evaluate whether the difference in healthcare costs found in this study was attributable to chance.

In conclusion, although twice-weekly psychotherapy sessions result in faster treatment response for depressive symptoms than once-weekly psychotherapy sessions at 6 months,^8^ there is no significant difference between the groups in the severity of depressive symptoms after 12 months of follow-up. The willingness-to-pay per additional unit of effect should be quite high to reach an acceptable probability of cost-effectiveness for all included outcome measures. Therefore, we conclude that twice-weekly sessions of psychotherapy for depression are not cost-effective over the long term, based on the current results.

## Data Availability

The data that support the findings of this study are available from the corresponding author, J.E.B., upon reasonable request.
